# Perception of information to Swedish melanoma patients in routine clinical practice – a cross-sectional survey

**DOI:** 10.1186/s12885-022-09208-w

**Published:** 2022-02-09

**Authors:** Helena Tufvesson Stiller, Rasmus Mikiver, Srinivas Uppugunduri, Marcus Schmitt-Egenolf

**Affiliations:** 1 Department of Public Health and Clinical Medicine, Dermatology, Umeå University, Umeå, Sweden; 2 Regional Cancer Centre South East, Linköping, Sweden; 3 Department of Biomedical and Clinical Sciences, Linköping University, Linköping, Sweden

**Keywords:** Cancer, Information, Melanoma, Oncology, Quality of life, Patient reported outcome measures

## Abstract

**Background:**

Comprehensible, relevant information empowers patients, allowing them to take an active part in their care. We aim to investigate how Swedish melanoma patients perceive information provided in routine clinical practice and explore the correlation between satisfaction with information, symptoms and functioning scales, and quality of life.

**Methods:**

A cross-sectional study where EORTC QLQ-C30, EORTC QLQ-INFO25 and EQ-5D-3L questionnaires were sent to 1213 patients by post and 792 responded (65%).

**Results:**

Only 0.5% reported that they wished to have received less information. The amount of information received and the satisfaction with that information was age-dependent, where older patients reported receiving less information than younger patients. Middle-aged patients were more satisfied with the information compared to both younger and older patients. The perception of having received sufficient information correlated negatively with anxiety. Higher satisfaction with the information also correlated positively with scores for functioning scales and negatively with degree of symptoms. No difference was perceived in information levels between disease stage apart from the scale “information about other services”, where patients with more severe disease reported receiving more information. Men and women reported equal satisfaction concerning the information received.

**Conclusions:**

Patients lack sufficient information and age affects the perception of it. It is of utmost importance to tailor the information to suit the individual needs of a given patient, as satisfaction with the information received correlates with the patient’s well-being.

## Background

Effective communication is key to patient-centred cancer care. Comprehensible, relevant information empowers patients, allowing them to take an active part in their care. Informed patients report better quality of life [1, 2] and higher satisfaction with care [1], while unmet information needs correlate with increased risk of anxiety and depression [2] and increased fear of cancer recurrence [3, 4].

The incidence of cutaneous malignant melanoma has increased rapidly during the past decades. In Sweden, the increase rate is approximately 5% each year. [5] Melanoma, like in other oncologic diseases, has also shifted towards being more of a chronic disease. As such, and even after treatment is completed, cancer patients may still struggle with psychological, physical and emotional issues [4, 6, 7]. Melanoma patients are a heterogeneous group and notable differences can be seen based on age and sex [8]. Treatment regimens also vary considerably in relation to the stage of disease [9]. The needs are therefore likely to differ between subgroups of patients and we have previously been able to show that tumour thickness influences quality of life as well as function and symptoms [10]. The psychosocial needs of melanoma patients, need to be studied further [11] to find ways of improving their quality of life.

In this population-based study, we investigate how the perception of information given in routine clinical practice differs between subgroups of melanoma patients. We explore furthermore if the satisfaction with information is connected to function, symptoms and quality of life.

## Patients and Methods

### Study design

We performed a cross-sectional survey employing a questionnaire covering quality of life, information and symptoms. The study was approved by the local ethics committee in Umeå, Sweden (approval no 2014/58-31) and is in accordance with the declaration of Helsinki. Paper questionnaires, a return envelope and an information letter were mailed to patients during 2015 as described previously [10]. A reminder letter and a new paper questionnaire were sent after a month when necessary. The questionnaires reached the patients 9-15 months after melanoma diagnosis.

### Instruments

The European Organization for Research and Treatment of Cancer Quality of Life Questionnaire Core 30 version 3.0 (EORTC QLQ-C30 v3.0) consists of 30 questions divided into 9 multi-item scales: 5 functional scales - physical, role, cognitive, emotional, and social function; 3 symptom scales - fatigue, pain, nausea & vomiting as well as a global health and quality of life scale. The single items dyspnoea, insomnia, appetite loss, constipation, diarrhoea and financial problems are also included. The questionnaire has been translated and validated in a Swedish setting [12].

The EORTC Information Module (QLQ-INFO25) consists of 25 questions divided into 4 scales concerning information about the disease (4 items), medical tests (3 items), treatment (6 items) and other services (4 items) as well as 8 single items [13]. All analysis of data is performed according to the guidelines previously published in the EORTC QLQ-C30 Scoring Manual [14]. In addition, we also used 5 generic questions regarding the health-related quality of life according to the EQ-5D-3 L. These cover the areas mobility, self-care, usual activities, pain/discomfort and anxiety/depression [15].

### Patient population

The patient population has been described previously [10]. Briefly, patients were considered eligible if they were diagnosed with cutaneous malignant melanoma during 2013-2014, residing in the south-eastern or northern region of Sweden, were over 18 years old and registered in the Swedish melanoma register. The two regions were chosen based on accessibility of data. 1308 patients were identified from the register out of which 95 patients were excluded; 86 had deceased, 4 had moved to other parts of Sweden, 4 had emigrated, and 1 had an unknown address. The remaining 1213 patients were included. Patients consented to the study by sending in the questionnaire. The data generated during the current study are not publicly available as Swedish law does not allow the publication of personal data from the register, but a selection of parameters can be acquired from the Swedish melanoma register on request and upon ethical approval.

### Variables and Statistics

 All scores of the EORTC QLQ-C30 and QLQ-INFO25 questionnaires were transformed to a value between 0 and 100 according to the EORTC guidelines. Higher scores for the global health and quality of life, functioning scales, information scales and information single items are positive as they represent a higher level of functioning or information. In contrast, higher scores are negative for the symptom scales, as they represent a greater degree of symptoms. Results are reported as mean. The Mann-Whitney test was used to compare groups in different scales. The tests were two-sided and a p-value of <0.05 was considered statistically significant. All statistical analyses were performed using SPSS version 25 software (IBM, Armonk, NY, USA) or R version 3.5.1. (R Core Team, Vienna, Austria). Missing data was handled by pairwise deletion.

## Results

A total of 792 patients responded out of 1213 included (response rate = 65.3%). The responders were representative for the study population in terms of region of residence, sex, age, tumour site, histological subtype, tumour thickness, tumour ulceration, stage and extended surgery of the primary tumour (Table 1). 78% of the questionnaires were complete, 10% were missing an answer to one question and 12% were missing answers to two or more questions. Since not all respondents answered all questions, the number of respondents varies between scales and items as noted in the figures.

**Table 1:** Background parameters for the responders and study population

	Respondents		Study population	
	(*n* = 792; 65.3%)		(*N* = 1213)	
	Men	Women	Men	Women
**Region (n, (%))**				
South East	273 (70.2)	281 (69.7)	406 (68.6)	417 (67.1)
North	116 (29.8)	122 (30.3)	186 (31.4)	204 (32.9)
**Sex (n, (%))**	389 (49)	403 (51)	592 (49)	621 (51)
**Age (mean)**	65	59	64	59
**Tumour site (n, (%))**				
Head/neck	54 (13.9)	33 (8.2)	79 (13.3)	54 (8.7)
Upper extremity	77 (19.8)	87 (21.6)	128 (21.6)	134 (21.6)
Lower extremity	50 (12.9)	145 (36)	69 (11.7)	207 (33.3)
Trunk	200 (51.4)	131 (32.5)	308 (52)	217 (34.9)
Hand, foot and subungual	4 (1)	6 (1.5)	4 (0.7)	8 (1.3)
Missing	4 (1)	1 (0.2)	4 (0.7)	1 (0.2)
**Histopathological subtype (n, (%))**				
Superficial Spreading Melanoma	260 (66.8)	268 (66.5)	398 (67.2)	418 (67.3)
Lentigo Maligna Melanoma	17 (4.4)	14 (3.5)	28 (4.7)	23 (3.7)
Nodular Melanoma	50 (12.9)	57 (14.1)	75 (12.7)	86 (13.8)
Acral Lentiginous Melanoma	3 (0.8)	6 (1.5)	6 (1)	9 (1.4)
Other	51 (13.1)	45 (11.2)	66 (11.1)	65 (10.5)
Missing	8 (2.1)	13 (3.2)	19 (3.2)	20 (3.2)
**Tumour thickness (n, (%))**				
<=1.00 mm	210 (54)	230 (57.1)	321 (54.2)	363 (58.5)
1.01-2.00 mm	92 (23.7)	81 (20.1)	136 (23)	111 (17.9)
2.01-4.00 mm	53 (13.6)	49 (12.2)	74 (12.5)	78 (12.6)
>4.00 mm	31 (8)	40 (9.9)	58 (9.8)	66 (10.6)
Missing	3 (0.8)	3 (0.7)	3 (0.5)	3 (0.5)
**Tumour ulceration (n, (%))**				
Present	62 (15.9)	62 (15.4)	99 (16.7)	97 (15.6)
Absent	309 (79.4)	320 (79.4)	461 (77.9)	493 (79.4)
Missing	18 (4.6)	21 (5.2)	32 (5.4)	31 (5)
**Stage (n, (%))**				
I	277 (71.2)	287 (71.2)	423 (71.5)	445 (71.7)
II	76 (19.5)	81 (20.1)	122 (20.6)	132 (21.3)
III –IV	33 (8.5)	30 (7.4)	42 (7.1)	39 (6.3)
Missing	3 (0.8)	5 (1.2)	5 (0.8)	5 (0.8)
**Extended surgery of the primary tumour (n, (%))**				
Yes	344 (88.4)	362 (89.8)	509 (86)	542 (87.3)
No	35 (9)	37 (9.2)	71 (12)	70 (11.3)
Missing	10 (2.6)	4 (1)	12 (2)	9 (1.4)

### Satisfaction with information in relation to quality of life and symptoms

To understand how information perception is connected to quality of life, we linked the results from QLQ-INFO25, the questions from EQ-5D-3L and the previously published QLQ-C30 [10].

**Fig. 1:**
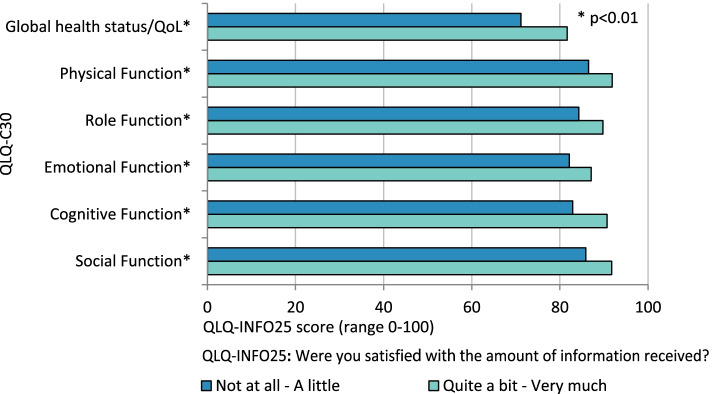
Results for EORTC QLQ-C30 in relation to satisfaction with information. Scales and items from EORTC QLQ-C30 presented by response to the question “Were you satisfied with the amount of information received” from EORTC QLQ-INFO25. A high index represents good function. * Significant difference between satisfaction with the information received (Not at all – A little and Quite a bit – Very much) for Global Health Status (*p* < 0.001), physical- (*p* < 0.001), role- (*p* = 0.004), emotional- (*p* < 0.001), cognitive- (*p* < 0.001) and social function (*p* < 0.001)

The perception of having received sufficient amount of information correlated positively with scores on function scales and quality of life (Fig. 1), showing that patients who are well informed experience a higher degree of functioning.

Significant differences between patients who were satisfied with information compared to those not satisfied could be seen for all function scales: physical (*p* < 0.001), role (*p* = 0.004), emotional (*p* < 0.001), cognitive (*p* < 0.001) and social function (*p* < 0.001); For the Global Health Scale (*p* < 0.001) and the symptom scales with the items fatigue (*p* < 0.001), pain (*p* < 0.001), dyspnoea (*p* = 0.001), insomnia (*p* = 0.003), appetite loss (*p* < 0.001), constipation (*p* = 0.005) and diarrhoea (*p* = 0.003).

**Fig. 2:**
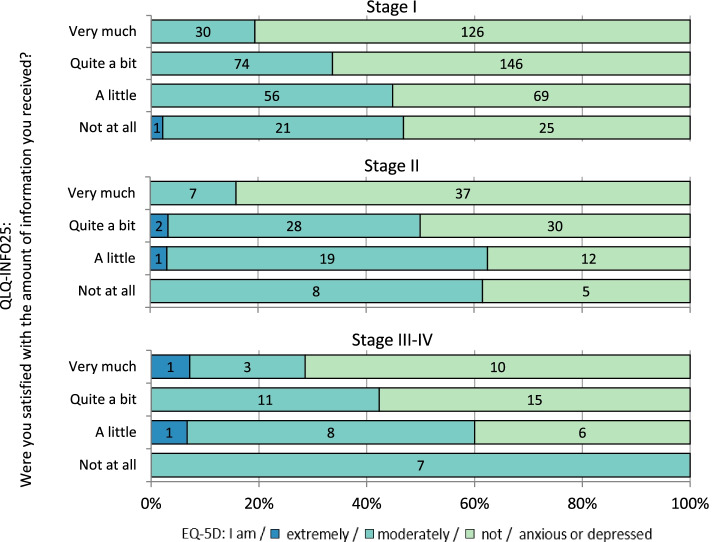
Satisfaction with information in relation to Anxiety/Depression. Results for the question “Were you satisfied with the amount of information received” from EORTC QLQ-INFO25 in combination with the dimension Anxiety/Depression from EQ-5D-3L. Results are presented by cancer stage. Numbers inside the bars indicate number of patients in each group. A Spearman correlation of -0.250 was significant (*p* < 0.001) for all groups combined. Spearman correlation for stage I:-0,216 *p* <0.001, stage II:-0,334, *p* <0.001 and stage III-IV:-0,373, *p* = 0.004

In a similar manner, the perception of having received sufficient amount of information correlated negatively with anxiety or depression (*ρ*=-0.250, *p* < 0.001) (Fig. 2).Thus patients who feel uninformed are at higher risk for anxiety and depression.

### Age dependency

**Fig. 3:**
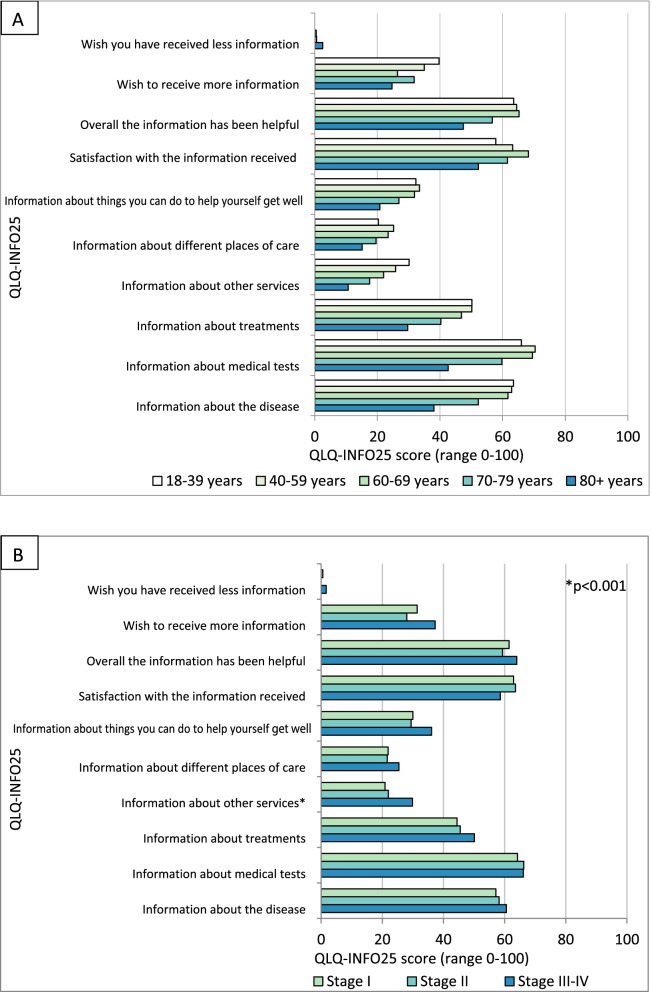
EORTC QLQ-INFO25 presented by Age (panel A) and Stage (panel B). **A**: Middle aged patients report receiving more information compared to both younger and older patients. Patients aged 80+ report receiving the least amount and lowest satisfaction with information. Patients in all age groups score information related to medical tests, the disease and treatments high, indicating they are feeling informed about these areas, while the categories “information about things you can do to help yourself get well”, “information about different places of care” and “information about other services” are scored lower, indicating patients are feeling less informed. 18-39 *n* = 64±1, 40-59 *n* = 240±6, 60-69 *n* = 204±12, 70-79 *n* = 167±9, 80+ *n* = 66 ± 9. **B**: Patients of all disease stages report similar levels of information in all areas except “Information about other services” where there is a statistically significant difference between patients with stage I-II and III-IV. Stage I *n* = 536±27, Stage II *n* = 142±9, Stage III-IV *n* = 59±3. * Significant difference between stage I-II and III-IV (*p* < 0.001)

We found that the amount of information received was age-dependent, where older patients reported receiving less information than younger (Fig. 3 A).

Patients aged 40-69 years reported receiving most information while patients aged 80+ reported receiving the least amount. The oldest patients (80+) were least satisfied with the information given and felt the least need to receive more information. Only 0.5% of patients wanted to receive less information. Middle-aged patients (40-79) were more satisfied with the information compared to both younger and older patients (Fig. 3 A). Men and women reported similar satisfaction with information received.

### Stage dependencies

We detected no difference in information levels perceived based on disease stage apart from the scale “information about other services” where patients with more severe disease reported receiving more information (*p* < 0.001) (Fig. 3B).

## Discussion

We present here a cross-sectional population based study, using internationally validated questionnaires where melanoma patients were selected based on the region of residence and year of diagnosis alone. We provide a representative population of patients with respect to melanoma type, stage, age as well as other relevant parameters. Better functioning and less symptoms correlated with higher satisfaction with the amount of information received. Further, older patients were less satisfied with information compared to middle-aged patients. Information is perceived equally across tumour stages. Taken together, patients feel insufficiently informed and this problem spans across several areas.

### Satisfaction with information in relation to functioning and anxiety

Melanoma patients report a high quality of life when compared to the general public [4, 16], but there are differences within this patient group. Patients with thin melanomas (≤ 2 mm) report functioning and quality of life on similar levels as the healthy public while patients with thicker melanomas report lower levels of functioning on several scales as well as lower quality of life [10] .

We show here that satisfaction with information is a second factor correlating with functioning and quality of life. Higher satisfaction with the information correlates positively with functioning and negatively with degree of symptoms. We found that in patients unsatisfied with the amount of information, functioning and quality of life is lower compared to satisfied patients, regardless of disease stage. Satisfaction with information correlated negatively with anxiety and depression which is in line with both earlier cross-sectional [4] and longitudinal [2, 17] studies. We were unable to investigate causality in our study due to lack of longitudinal data. However, our results are compatible with the pattern described in earlier studies. The relationship between satisfaction with information and anxiety has been proven by others to be bidirectional, where satisfaction with information at baseline predicts anxiety levels after a year as well as vice versa [17]. Other studies have shown that the level of melanoma associated anxiety and depression is higher at the beginning of treatment, diminishing over time [18] and settles at a level slightly above the general population in long-term survivors [19]. Even though our study was conducted several months after diagnosis, there was still a large proportion of patients with anxiety/depression problems, indicating that this is an area where better support from the health care system would be of great benefit. Since patients with metastatic melanoma treated with immunotherapy are at an increased risk of emotional distress including anxiety and depression [20, 21], there is a significant unmet need for proper support and information in this group. The lack of sufficient information causing anxiety also extends to informal caregivers of cancer patients [22]. Previous studies suggest that providing patients with proper information can prevent anxiety and depression later on [2, 17]. Thus, providing patients with adequate information would be of potential benefit for the patients, caregivers and the health care system.

### Unmet information needs

A German study showed that as many as 25% of the patients felt they had received too much information [22]. In our study, only 0.5% reported that they wished to have received less information. This discrepancy may be caused by differences in culture [24], patient population, or the actual amount of information provided. Patients reported similar low amounts of information received as well as similar low satisfaction with that information across all disease stages in the current study. Similarly, an Australian study report unmet information needs in 40-50% of melanoma survivors [4]. The low number of patients that reported receiving too much information in our study indicate that patients are open to receiving more information.

### Age and gender dependencies

The amount of information received and the satisfaction with that information was age-dependent, where older patients reported receiving less information than younger. Middle-aged patients are more satisfied with the information compared to both younger and older patients. Hence, melanoma patients fit into the pattern described for other types of cancer [24]. This may be related to differences in preferences for different sources of information between older and younger melanoma patients and the way information is provided to them [25, 26]. It is possible that health care workers and patients that are similar in age may communicate more efficiently. This could potentially explain the higher satisfaction with information in 40-69 year old patients seen in our study. As shown previously, younger individuals may be more inclined to search for information on the internet themselves compared to older patients. The most satisfying information however, is the one provided by health care staff [25]. In line with Brütting et al. [23], we also found lower levels of received information and satisfaction with information in elderly individuals. Interestingly, elderly patients do not wish to receive more information compared to other groups. As suggested previously [27], this may depend on elderly patients being primarily interested in the disease and its effects and being less interested in detailed information. It is also possible that younger relatives take on a care giver role and, thus, assume responsibility for the information. This could explain why older individuals feel less informed but still do not request for additional information.

In all age groups, patients feel more informed in areas related to the disease and its treatment and less informed in areas related to other places of care, self-help and other services. This may be explained by the fact that information about the disease and treatment is relevant to all patients, while information about other services and different places of care may only be relevant for patients with a more severe disease.

Men and women reported equal satisfaction concerning the received information in our study. In contrast, a German study [23] showed that women often felt less informed compared to men in areas like information about the disease, diagnostics and psychological support.

### Limitations of the study

A longitudinal design could have provided insights relating to causality between anxiety and satisfaction with information. The questionnaires were sent 9-15 months after diagnosis which means severely ill patients may have deceased before the start of our study. It is also possible that patients may have forgotten some of the information that was given to them earlier in the process.

We lack detailed knowledge regarding the information provided to these patients. Further, it is, not only possible that patients may have received different information but also that they may interpret the same information differently.

As in many surveys, responder bias must be considered. Individuals with strong opinions are more likely to respond compared to individuals with less strong opinions.

### Clinical Implications

The information needs of melanoma patients and the ability of the health care system to meet those needs are of great importance for the physical and mental well-being of the patient. The need for information is most likely universal, but individual needs may vary, depending on factors such as age and capabilities. Health care practitioners should therefore prioritise personalized information as part of routine practice.

### Future research

It remains to be studied if improving information in unsatisfied patients will reduce the risk of developing anxiety or depression. Finding a suitable time point for measuring base line anxiety and depression could possibly improve satisfaction with information further on. It also remains to be studied if different ways of delivering information has any impact on the patients’ well-being. There are relatively few studies on the connection between information and quality of life. As cancer is becoming more of a chronic condition and anxiety is an increasing problem, we need to understand how these questions may be addressed in order to give patients a better quality of life.

## Conclusions

It is of utmost importance to tailor the information towards the individual needs of a given patient, as the satisfaction with the information received impacts the patient’s well-being. Demographic factors such as age needs to be taken into account. Care givers should therefore focus on addressing this unmet information need in melanoma patients.

## Data Availability

The data that support the findings of this study are available from the Swedish melanoma register but restrictions apply to the availability of these data, which were used under license for the current study, and so are not publicly available. Data are however available from the authors upon reasonable request and with permission of the Swedish Melanoma Register.
